# Eye, Ear, Nose, and Throat

**Published:** 1896-01

**Authors:** 


### EYE, EAR, NOSE, AND THROAT.

EDITED BY DUNBAR ROY, A.B., M.D.

### THE TREATMENT OF TINNITUS AURIUM.

A valuable report upon this subject was presented by Drs. Meol and Herck at the last meeting of the French Society of Otology and Laryngology.

The authors suggest the following classification:

1. Entotic or periotic noises generally perceptible by both physician and patient.

2. Tinnitus proper, noises and sensations without apparent acoustic cause.

*A.*—Tinnitus due to a lesion of the auditory apparatus:

1. In diseases of the external ear.

2. In diseases of the middle year.

3. In diseases of the internal ear.

*B.*—Tinnitus compatible with integrity of the auditory apparatus:

4. In diseases of the nervous system.

5. In mental diseases.

6. Reflex.

It is very generally recognized by otologists that treatment of the tinnitus alone is very often ineffective, and that a preliminary investigation into the cause of the trouble, whether depending upon systemic disease or a local condition is not merely valuable but necessary. The diseases of the ear itself which frequently cause tinnitus are first considered: I. Tinnitus in affection of the external ear may be due to: (1) Hyperemia of the soft tissues of the meatus and drumhead, or by reflex action on the muscles of the middle ear, on the branches of the auditory nerve, and (2) obliteration or hyperemia of the meatus by cerumen, etc., with consequent pressure on the drumhead and later on the perilymph and reflexly on the labyrinth; (3) inflammation of the external meatus furuncle, parasitic otitis, syphilitic otitis, etc. The treatment in these cases is frequently effective.

II. *Tinnitus in Affections of the Middle Ear, etc.* In affections of the tympanic cavity and eustachian tube the tinnitus may depend upon :

(1) An inflammatory condition of the mucous membrane capable of producing a hyperemia of the labyrinth. This can be modified advantageously by cold applications and bleeding (leeches), by inflation of the middle ear and removal of secretion if any is present (2) a subacute or chronic catarrhal condition, with or without tubal obstructions. The potency must be restored if obstruction exists; absorption of the products of inflammation facilitated where possible by inflation of the middle ear, and paracentesis followed by irrigation of the tube and tympanic cavity practiced when necessary. In narrowing of the eustachian tube bougies may be employed; when its lumen is obliterated the cicatrinal tissue may be destroyed with the galvano-cautery, or it may be perforated, or a permanent perforation of the tympanic membrane produced; (3) in sclerosis or dry otitis media the best results are secured with the "masseur" of Delstanche, intratympanic injections (iodine in vaseline) and counter-irritation over the mastoid process. When the tinnitus appears to depend upon the existence of synechiæ, retractions, anchylosis, the appropriate operation as myringectomy, section of the anterior or posterior fold, tenotomy of tensor tympanic or stapedius, mobilization of the stapes, removal of one or more ossicles may be attempted, but the authors agree with the majority of otologists in believing that good effects, when secured at all, are not permanent. Among medicaments may be mentioned potassium bromide in large doses, particularly in neurasthenic cases; quinine alone or with iron, iodide of potassium (in syphilis); as instillations: hot water, ether, glycerine, particularly the last, in extreme dryness of the drumhead external meatus; hypodermically: morphine, strychnine and pilocarpine; (4) when the tennitus is due to an otorrhea with persisting perforations authors recommend the constant current, six or eight milliamperes for five to ten minutes, the positive pole over the auricle, the negative at the back of the neck. Counter-irritation over the mastoid is also useful, but must be used with caution. (5) Noises having their origin in an affection of the tympanic membrane exclusively are comparatively infrequent and their extended consideration may be omitted in this notice.

Among the more important may be mentioned relaxation of the membrane, in which collodion gives the best results, if there is not too great an atrophy of the fibrous layers.

If the atrophy is advanced, artificial perforation may be tried. The noises co-existing with synechia exaggerated tension are much lessened sometimes by the application of collodion. Surgical interference is sometimes necessary, but when the tension is due to retraction of the membrane in dry otitis media has, as a rule, but a temporary effect.

III. *Internal Ear.*—The pathological condition of the labyrinth causing tinnitus are anemia, hyperemia, extravasation of blood, inflammation and chronic lesions of the labyrinth. (1) Anemia may be caused by general anemia or by the result of an aneurism of the basilar artery, atheroma of the internal auditory, etc.; (2) in hyperemia the cause must first be determined. If there is cerebral congestion and redness of the face, Leiter's tubes are indicated and a little later mastoid counter-irritation, intestinal derivatives, bromide and iodide of potassium, pilocarpine, galvanism applied to the upper cervical ganglion of the sympathetic. It must be remembered that the hyperemia may come from the administration of quinine, salicylic, etc.; (3) in hemorrhages into the semicircular canals, vestibule and cochlen, one may first employ the means already suggested in hyperemia, and when the active stage has disappeared (third week) pilocarpine subcutaneously. In Menier's disease, Charcot has used sulphate of quinine with success. This should be used for one fortnight only. In the treatment of (4) imflammation of the labyrinth occurring *e. g.* as a part of a panotitis in scarlet fever, and in (5) chronic hyperplasias and degenerations following hyperemias, hemorrhages, and imflammations of the part, but little of advantage can be suggested. The possibility of syphilis must always be considered.

IV. *Tinnitusin Diseases of the Nervous System..*—Among the affections in which it may exist are arterio-sclerosis, affecting the cerebral arteries, tumors, acute bulbar myelitis, tubes, cervico-occipital neuralgia, and in neuroses (epileptic and hysterical aura, neurasthenia, migrane).

V. *Tinnitus in Mental Diseases.*

VI. *Tinnitus of Reflex Origin is Unusual.*—Sufferers from stomach troubles, for example, have frequently a beginning sclerosis of the ear, and the subjective sounds so frequently noticed in connection with uterine affections may often be attributed to the anemia or neurasthenia of the patient. [The author seems to have overlooked a very important source for reflex aural noises in hypertrophies, and exostoses in the nasal chamber. I have found many cases of tinnitus aurium removed by the removal of these enlargements in the nasal chambers.—D. R.]—*Annals of Ophthalmology and Otology.*

